# Perceptions of perinatal alcohol use and treatment needs in Cape Town, South Africa: a qualitative study

**DOI:** 10.3389/fpsyt.2024.1199647

**Published:** 2024-03-13

**Authors:** Petal Petersen Williams, Lesley-Ann Erasmus-Claassen, Shantae Taylor, Felicia A. Browne, Wendee M. Wechsberg, Bronwyn Myers, Charles D. H. Parry, Yukiko Washio

**Affiliations:** ^1^ Alcohol, Tobacco and Other Drug Research Unit, South African Medical Research Council, Cape Town, South Africa; ^2^ Institute for Life Course Health Research, Department of Global Health, Stellenbosch University, Stellenbosch, South Africa; ^3^ Department of Psychiatry and Mental Health, University of Cape Town, Cape Town, South Africa; ^4^ Substance Use, Gender and Applied Research, Research Triangle Institute (RTI) International, Research Triangle Park, NC, United States; ^5^ Gillings School of Global Public Health, University of North Carolina, Chapel Hill, NC, United States; ^6^ Psychology, North Carolina State University, Raleigh, NC, United States; ^7^ Psychiatry and Behavioral Sciences, Duke University School of Medicine, Durham, NC, United States; ^8^ Curtin enAble Institute, Curtin University, Bentley, WA, Australia; ^9^ Department of Psychiatry, Stellenbosch University, Stellenbosch, South Africa; ^10^ Department of Obstetrics, Gynecology and Reproductive Sciences, Temple University Lewis Katz School of Medicine, Philadelphia, PA, United States

**Keywords:** alcohol use, pregnant women, breastfeeding, perinatal, South Africa

## Abstract

**Background:**

South Africa has one of the world’s highest rates of foetal alcohol spectrum disorders (FASD). Recent evidence also showed that alcohol use during lactation significantly compromises child development in children exposed to alcohol through breastfeeding, independent of prenatal alcohol exposure. This study explored perceptions of perinatal alcohol use and treatment needs in Cape Town, South Africa, to inform the development of an intervention to encourage alcohol abstinence during pregnancy and breastfeeding.

**Methods:**

Individual in-depth interviews (IDIs) were conducted with women who were pregnant with a recent history of alcohol use (n=32) and clinic and community stakeholders (n=16). Interviews were audio recorded and transcribed verbatim. Coding and thematic analyses were conducted in NVivo 12.

**Results:**

Results indicate widespread perception that women know the dangers of drinking alcohol while pregnant with much less known about drinking while breastfeeding. Mixed views were shared about whether women who are pregnant or breastfeeding experience alcohol-related stigma. Participants described contextual factors impacting drinking that include interpersonal violence, lack of support, stress, anxiety and poverty, and drinking being normalised. Finally, participants had mixed views and conflicting knowledge of available resources to support alcohol reduction and highlighted a desire for support groups and the involvement of partners in alcohol interventions.

**Conclusions:**

Findings from this study highlight the need for an alcohol intervention programme that is innovative and tailored to the needs of women who are pregnant or postpartum. It also highlights the importance of including community-based support and partner involvement in these interventions.

## Introduction

South Africa reports one of the highest per capita rates of alcohol consumption in the world (1, 2) of 9.3L of pure alcohol in 2016 (29.9L of pure alcohol per drinker) (3). In a nationally representative sample, current alcohol use in 2014-2015 was reported by 20% of women and among these women, 32% reported binge drinking (defined as consuming 4 or more drinks on an occasion for women) (4). Not surprisingly, South Africa has one of the world’s highest rates of alcohol-exposed pregnancies and foetal alcohol spectrum disorders (FASD) because of prenatal alcohol exposure (5–7). A meta-analysis found a prevalence of 13.2% of maternal alcohol use during pregnancy in South Africa (8), higher than the average rate in the WHO African region (10.0%) and globally (9.8%) (5). Binge drinking is particularly harmful during pregnancy, as the resultant rapid increases in blood alcohol concentrations (BACs) increase risk for FASD (9).

While many people reduce or stop drinking after becoming aware of their pregnancy, studies have shown that pre-pregnancy drinking levels are predictive of drinking levels during pregnancy (10). Additionally, even among those who stop during pregnancy, many will return to drinking post-partum. This is a concern as alcohol use during breastfeeding significantly compromises a child’s development independent of prenatal exposure (11). In South Africa, drinking while breastfeeding is more common among mothers who drink prenatally, and over 40% of mothers who abstained from prenatal drinking report alcohol use while breastfeeding (11).

Despite the strong evidence for the associations between prenatal and postpartum alcohol use and risk for FASD and compromised child development (11), there is no specific policy to prevent FASD in South Africa (12–14). Additionally, there are few feasible, effective, and sustainable interventions that address prenatal and postpartum drinking while breastfeeding (15–17). While South African studies have identified several factors to consider in developing interventions (18–23), little attention has been paid to women’s experiences, beliefs and perceptions of alcohol use and their treatment needs (24). This is important to ensure that potential beneficiaries of the intervention contribute to the intervention design in order to enhance acceptability, uptake and engagement down the line (25). Furthermore, a qualitative study previously highlighted the difficulties that health care providers in Cape Town, South Africa face in addressing alcohol use among pregnant women in antenatal care and the importance of giving voice to their concerns in the development of appropriate interventions (26). Finally a community survey to assess knowledge and attitudes to risky drinking and responses to policy options to address such practices (27) suggests that community stakeholders voices are also important. This qualitative study therefore aimed to explore perceptions of perinatal alcohol use and treatment needs in Cape Town, South Africa, from the perspectives of pregnant and breastfeeding women who drink, and clinic and community stakeholders. This research activity was conducted to assist in the development of an intervention to encourage alcohol abstinence during pregnancy and breastfeeding.

## Methods

### Setting and recruitment

This qualitative study is presented in line with COnsolidated criteria for REporting Qualitative research (COREQ) guidance (28). We conducted in-depth interviews with women who were pregnant and who reported alcohol use during the current pregnancy. Women were recruited from several communities in the Cape Flats region of the Western Cape Province of South Africa. Additionally, we conducted key informant interviews (KIIs) with clinic and community stakeholders providing services to women who are pregnant and postpartum, to explore their perceptions, experiences, and knowledge about perinatal alcohol use; breastfeeding while drinking; relevant health and psychosocial issues; necessary treatment and services to address these issues; and barriers to linking women to the required services.

For women who were pregnant and postpartum, community-based outreach techniques and snowball sampling were used to market the study and recruit participants. Field staff regularly visited areas frequented by potential participants to enhance visibility and build rapport with community members. To be eligible, women had to (1) be 18 years of age or older, (2) be currently pregnant or delivered within the last three months, (3) report drinking during the current pregnancy or while breastfeeding, (4) have their own mobile phone, (5) intend to breastfeed for at least 6 months, (6) be able to participate in the interview in English, and (7) voluntarily consent to participate in the study. Clinic and community stakeholders were recruited through two Midwife Obstetric Units (providing antenatal services to women) and a community collaborative board that our team has worked with in past studies.

### Procedures

Interviews were conducted by the second author (LEC) and a research assistant who were both female. LEC has a post-graduate degree and more than five years qualitative research experience. Prior to the interviews, participants were asked to provide written informed consent. Interviews with pregnant and postpartum women were conducted within a private space in the community and healthcare clinics. Interviews with clinic and community stakeholders took place at their place of work or telephonically. Interviews lasted up to 60 minutes. Interviews took place between July and November 2021. All interviews were audio-recorded and transcribed verbatim. Participants were reimbursed ZAR150 (~US $8.31 [ZAR18.04/$1]) for their time.

### Data analyses

NVivo 12 software was used to manage the qualitative data. Data were analysed thematically using the Braun and Clarke approach (29). We combined a deductive approach to coding based on the research questions with an inductive approach to allow for the identification of emergent themes. The first author (PPW) conducted the initial process of familiarization through a review of transcripts and coding. The first and second author (LEC) discussed the initial framework and individually coded the first two transcripts. Following this, they discussed refining of codes and themes. Intercoder reliability checks were conducted, with a Cohen’s Kappa score of 0.80 being obtained. Coding then continued independently for all transcripts. Any coding disagreements were resolved through discussion and consultation with the last author (YW).

### Ethical approval

Ethical approval to conduct the study was granted by the South African Medical Research Council’s Human Research Ethics Committee and the Western Cape Department of Health.

## Results

Three major themes were identified: perceptions and practices of alcohol use during pregnancy and breastfeeding; contextual factors impacting perinatal drinking; and perception of accessibility of resources to support women who drink.

### Sample characteristics

We conducted 32 interviews with women who were pregnant with a mean age of 26 years; 31 were Coloured (of mixed-race ancestry) and one participant identified as Black African. A total of 16 clinic and community stakeholders were interviewed. These consisted of researchers, government officials, substance use treatment specialists, clinic-based health care workers and community health workers.

### Perceptions and practices of alcohol use during pregnancy and breastfeeding

#### Knowledge about alcohol use during pregnancy and breastfeeding

There was an overall perception that women continue to drink during pregnancy. Most stakeholders and perinatal participants thought women were aware of the dangers of alcohol use in the perinatal period but continued to drink anyway with only a few thinking continued drinking was due to women being uneducated about the harms.

*It is very seldom that we nowadays find that women have never heard that alcohol can be detrimental to the baby.* (Stakeholder participant 1)

*Alcohol is dangerous for a child because of development for a baby and yes … It’s dangerous … It was explained at the clinic….everyone has that information that it’s dangerous for a child to take in alcohol but everyone does their own thing.* (Perinatal participant 1)

Perinatal participants were able to demonstrate their own knowledge of the harmful effects of alcohol on the foetus, with some sharing their experiences of how alcohol use affected birth outcomes of their own children. Contrary to this, misconceptions associated with maternal drinking exist particularly in cases where women drank alcohol previously and felt there was nothing wrong with their children. Specifically, 22 participants reported they knew the effects of alcohol on the foetus, while one specifically reported not knowing the effects. When speaking about others, comments from six participants suggested that they thought women in the community generally know the impact alcohol has on the foetus, while two thought women might know, five said with certainty that women in the community do not know and eight participant were unsure.

Stakeholders and perinatal participants confirmed widespread breastfeeding among mothers in their communities. While participants described widespread knowledge of the dangers of alcohol use while pregnant, they felt that women knew little about the effects of alcohol use when breastfeeding.

*The debate about breastfeeding and alcohol use is very new … And I think that must be added to all intervention programs and information sessions. Because a lot of women don’t realize that.* (Stakeholder participant 2)

#### Stigma related to alcohol use during pregnancy and breastfeeding

Participants shared very mixed views about whether stigma was experienced by women who drink alcohol while pregnant or breastfeeding. On the one hand some stakeholders and perinatal participants shared that these women are treated poorly and experience stigma:

*I don’t think they will get judged but I think maybe they will lose their friends and they might not be willing to do that.* (Stakeholder participant 6)

Contrary to this view, other participants thought that women who continue to drink alcohol during pregnancy or while breastfeeding were treated no differently from women who did not drink, because drinking during pregnancy and the postpartum period is normalised. In total, 14 perinatal participants and six stakeholders thought women who drink alcohol while pregnant or breastfeeding experience stigma, while six perinatal participants and three stakeholders thought this was not the case.

### Contextual factors impacting drinking during pregnancy and the postpartum period

Participants described various contextual factors influencing perinatal women’s drinking patterns, such as interpersonal violence, lack of familial and community support, stress and anxiety, poverty, and substance use as a way of life.

#### Interpersonal violence

Violence was described by both stakeholders and perinatal participants as being closely interlinked with alcohol use. Experiences of violence and other forms of abuse were described as the reason for why people drink, in order to forget and also because they feel isolated and unable to share their hardships with anyone. Second, it was thought that drinking in social environments where violence commonly occurs has resulted in the normalization of exposure to violence in the lives of people. In fact, it was pointed out that women can also be the aggressors or abusive towards men even when pregnant as they believe their partners won’t retaliate given their pregnancy. Finally, violence was also described as something that is sometimes experienced by perinatal women because of their drinking where women are assaulted because they are ‘drunk or unruly’.

#### Lack of support

Stakeholder and perinatal participants described a lack of familial and community support and lack of emotional, psychological and physical support from romantic partners as influencing drinking behaviour in perinatal women. While they mostly discussed how lack of partner support influenced their use of alcohol, perinatal participants also discussed the support they desire from services in the community.

*No, there’s no resources … for women in general when you’re pregnant or not … I’m alone at times when my husband is at work or wherever then I’m with her alone all the time. And it’s sometimes very hard … there’s no help for you … No help from police, no help from the clinics.* (Perinatal participant 5)

Stakeholders specifically talked about the lack of support perinatal women receive from their partners. They felt that men often believe their role is complete after conception but that they in fact play a big role during pregnancy and after. These participants felt that male partners could play more of a role in supporting their partners to change their alcohol use by reducing their own drinking. Additionally, the pressures and stressors women experience (such as lack of financial support or support with their babies) from these men often lead their partners to drink, feeling trapped in those situations.

#### Stress, anxiety and poverty

Stakeholders and perinatal participants described the co-occurrence of mental health difficulties and alcohol use among pregnant or breastfeeding women. According to one participant, these difficulties included depression, stress, and psychological trauma.

*I think some of them come out of broken homes. Stuff that happened in the past. Childhood injuries, childhood trauma and all that. So, it’s a lot of different reasons why they drink. But now they don’t drink to just drink, but they drink to solve that problem by creating another problem on top of that … Some of it is very, very bad.* (Perinatal participant 20)

Financial difficulties were raised as a prominent stressor, with participants reflecting that women may use alcohol to forget their circumstances. Perinatal women specifically mentioned high levels of unemployment and poverty in their communities. Stakeholders also described how extreme poverty in these communities led to social stress, with alcohol being used to forget this stress.

#### Alcohol as a way of life

Both stakeholders and perinatal participants described alcohol use during pregnancy and breastfeeding as a way of life in these communities. They noted that alcohol use (including binge drinking) was part of the community culture particularly over weekends where it was viewed as a means of recreation. According to perinatal participants, this culture of binge-drinking over weekends contributed to drinking in pregnancy and while breastfeeding being normalized.

*I think, alcohol use over weekends, it’s such an ingrained way of life. People really don’t know what else to do with their time*. (Stakeholder participant 1)

*Well, in our community that’s - that is something usual … Because everybody does it … Everyone is drinking while they’re pregnant.* (Perinatal participant 15)

### Perceptions of accessibility of resources to support women who drink

#### Existing resources are limited

Participants had mixed views and conflicting knowledge of available resources for perinatal women who drink alcohol. While some thought there were alcohol intervention support services available, many felt there was nothing available beyond what is offered at antenatal clinics. Among perinatal participants, nine indicated that clinic services were the only resource available while seven stakeholders reported the same. Three stakeholders were however able to describe services beyond the antenatal clinics. Fifteen perinatal participants were unsure or unaware of resources for help or information in their communities. Stakeholders also pointed out that not all primary healthcare clinics where antenatal services are provided had equal available resources to offer support.

The majority of perinatal participants responded ‘yes’ when asked if the antenatal clinic provides information about effects of alcohol (and other drug) use when pregnant. However, it was evident that much less information was available around alcohol use and breastfeeding. Beyond the primary healthcare setting, stakeholders pointed out that alcohol treatment services were provided by non-government organisations.

*Now the problem with that is that not all clinics have got a mental health nurse, because addictions falls under mental health. So you end up referring the person to maybe an NGO or something like that.* (Stakeholder participant 3)

#### Desired additional resources

Perinatal participants expressed a desire for support groups that could be an additional resource for them in the community. In addition to support groups and the need to find ways to provide information about when these services are being offered and how to access them, both stakeholders and perinatal participants raised the importance of including partners in services or interventions for perinatal women who drink.

## Discussion

This study described perceptions about perinatal alcohol use and treatment needs in Cape Town, South Africa and highlighted the need for tailored interventions for pregnant and postpartum women. More specifically, findings highlight that knowledge about the dangers of drinking during pregnancy is widespread, with perinatal women being aware of the harm drinking can cause to their unborn foetus. This is in keeping with international literature which demonstrates widespread knowledge about the specific effects of alcohol consumption in pregnancy (30–32), but in contrast to a single SA study. In a study conducted in Cape Town participants were largely unfamiliar with FASD, and their knowledge of the impact of prenatal alcohol exposure was often inaccurate (33).

In this focused local study, personal experiences of alcohol use appeared to influence beliefs about the harms of alcohol during pregnancy. This can lead to misinformation with health implications beyond the woman. This suggests the need for interventions that not only focus on information sharing, but move beyond education to address women’s context and attitudes towards anti-drinking messages in order for them to feel heard and be more valued (33, 34).

Innovative approaches are required to help women understand the impact of their drinking on their unborn babies. One caveat of this is that women need to be enabled to become aware of their pregnancies much sooner given the high rates of unintended pregnancies (35) and the critical first stage of pregnancy and alcohol’s impact during this earlier stage. Even among women who stop or reduce drinking upon pregnancy recognition, heavy drinking and lack of appropriate vitamins such as folic acid prior to becoming aware of the pregnancy may impact the development of the foetus. Earlier interventions focusing on decreasing alcohol exposed pregnancies are thus crucial to optimize the health of women and their infants.

Findings from this study highlight that far less is known about the impact alcohol may have on breastmilk and breastfed infants. This may be because there is an abundance of evidence for the effects of alcohol use during pregnancy with robust guidelines outlining recommendations, but a paucity of scientific evidence about drinking during breastfeeding and subsequently few recommendations (36). The data thus revealed a need to educate women about harms associated with alcohol consumption while breastfeeding in light of the recent evidence (11), particularly harms around binge drinking among women who are still breastfeeding. There is also a need for more local research on this, to provide compelling local evidence and stories for women so they are comfortable that the evidence that they are presented with is locally relevant and relatable. Therefore, local evidence, stories and narratives need to be communicated to women to convince them of the relevance and suitability of the research evidence for their own lives and context.

Findings from this study also highlight mixed views about whether alcohol use while pregnant or breastfeeding is stigmatized. While some participants thought stigma was experienced, the overwhelming view was that those who do drink during the perinatal period were not treated any differently as drinking during pregnancy and breastfeeding was normalized in many communities. Given the high levels of alcohol consumption in South Africa including among women, and the particular culture of binge drinking, messages to remain abstinent while pregnant and breastfeeding often contradict social norms (33). Intervention programmes therefore need to focus on providing strategies that may lead to changes in how social norms are understood so that abstinence messages are not seen as contradictory to social norms but rather seen as necessary for the health of their infants. Interventions that address community and social norms around alcohol use in other settings by utilizing community reinforcement, advertising or social marketing, social mobilization and broader community-based alcohol prevention programs have shown to impact positively on drinking behaviour (37–41). Similarly, given the findings in the current study, interventions addressing community and social norms around alcohol use in the local context may impact on women’s drinking during the perinatal period.

Finally, this study highlights contextual factors influencing drinking which include experiences of violence, lack of support, stress, anxiety and poverty. Experiences of violence in pregnancy is consistent with research in Cape Town which found that as much as 15% of pregnant women had experienced intimate partner violence ranging from sexual and physical to emotional and verbal (42). The high level of violence during pregnancy was associated with poverty-related factors including food insecurity, mental ill-health, unemployment, unwanted pregnancies and past experiences of abuse (42). Local research has also shown that experiencing violence or aggression is a risk factor for alcohol use in pregnancy (19, 20). Additionally, depression, anxiety, suicidality, food insecurity, relationship dynamics, and past mental health problems were predictors of alcohol and other drug use (19), which supports our findings. In keeping with other studies of women who use alcohol in this setting (43, 44) findings demonstrate that alcohol was used as a way of coping with or managing these stressors and traumatic experiences and emotions.

Mixed views about accessibility of resources and support for alcohol use were also identified with the majority believing there are no resources beyond information provided at primary healthcare. The lack of support and in particular partner support influencing drinking behaviour is consistent with other studies which found that lack of partner support featured as a major factor in pregnant women’s drinking (45). Similar to previous studies with women in this setting (43, 44), our findings thus support the need for interventions to address intimate partner violence and associated mental health needs of women who are pregnant and breastfeeding, and to develop community-based networks such as support groups for those who want to reduce drinking. Evidence of partner co-dependency and traditional gender norms in relationships (45) supports the recommendation for couple interventions to include partners with regard to prenatal care, breastfeeding, and drinking.

Findings from this study should be considered in light of its limitations. Firstly, participants who were pregnant were recruited from a mix of communities in the Cape Flats region of the Western Cape province of South Africa. Therefore, the extent to which findings are representative of the total population of pregnant women who use alcohol in South Africa is unknown. Secondly, there may have been social desirability bias particularly from clinic and community stakeholders who may be invested in seeing a programme developed to prevent drinking in pregnancy and postpartum.

Despite these limitations, findings from this study support plans to develop an intervention programme that is relevant to the needs of women who are pregnant and breastfeeding who drink alcohol. It highlights the importance of developing intervention programmes that move beyond education about the harms of drinking in pregnancy to include people’s attitudes towards drinking in pregnancy. Second, it highlights the need to provide education around the harms associated with alcohol use and breastfeeding. Third, there is a need to focus on the social norms around alcohol use and the normalization of drinking among perinatal women. It highlights the importance of including community-based support such as peer support groups for perinatal women, referral for intimate partner violence and mental health support and inclusion of partners in the form of couple interventions to enable a supportive environment for having a healthy pregnancy and a thriving child.

## Data availability statement

The raw data supporting the conclusions of this article will be made available by the authors, without undue reservation.

## Ethics statement

The studies involving humans were approved by South African Medical Research Council’s Human Research Ethics Committee. The studies were conducted in accordance with the local legislation and institutional requirements. The participants provided their written informed consent to participate in this study.

## Author contributions

PP and YW conceptualized the study. L-AE-C conducted the qualitative interviews. PP, L-AE-C, ST and YW conducted the analysis and PP prepared the manuscript first draft. FB, BM, WW and CP helped develop and refine the study and all authors revised the draft versions of the manuscript critically. All authors contributed to the article and approved the submitted version.

## References

[1] Martinez P Røislien J Naidoo N Clausen T. Alcohol abstinence and drinking among African women: data from the World Health Surveys. BMC Public Health. (2011) 11:160. doi: 10.1186/1471-2458-11-160 21392398 PMC3061917

[2] Culley CL Ramsey TD Mugyenyi G Kiwanuka GN Ngonzi J Macleod S. Alcohol exposure among pregnant women in sub-saharan Africa: a systematic review. J population Ther Clin Pharmacol = J la therapeutique Des populations la pharamcologie clinique. (2013) 20:e321–33. 24163173

[3] World Health Organisation. Global status report on alcohol and health 2018. World Health Organisation: Geneva (2018).

[4] Vellios NG Van Walbeek CP. Self-reported alcohol use and binge drinking in South Africa: Evidence from the National Income Dynamics Study, 2014 - 2015. South Afr Med J = Suid-Afrikaanse tydskrif vir geneeskunde. (2017) 108:33–9. doi: 10.7196/SAMJ.2017.v108i1.12615 29262976

[5] Popova S Lange S Probst C Gmel G Rehm J. Estimation of national, regional, and global prevalence of alcohol use during pregnancy and fetal alcohol syndrome: a systematic review and meta-analysis. Lancet Global Health. (2017) 5:e290-e299. doi: 10.1016/S2214-109X(17)30021-9 28089487

[6] Lange S Probst C Gmel G Rehm J Burd L Popova S. Global prevalence of fetal alcohol spectrum disorder among children and youth: A systematic review and meta-analysis. JAMA pediatrics. (2017) 171:948–56. doi: 10.1001/jamapediatrics.2017.1919 PMC5710622 28828483

[7] Adnams CM. Fetal alcohol spectrum disorder in Africa. Curr Opin Psychiatry. (2017) 30:108–12. doi: 10.1097/YCO.0000000000000315 28125440

[8] Popova S Lange S Probst C Shield K Kraicer-Melamed H Ferreira-Borges C. Actual and predicted prevalence of alcohol consumption during pregnancy in the WHO african region. Trop Med Int Health. (2016) 21:1209–39. doi: 10.1111/tmi.12755 27429168

[9] Maier SE West JR. Drinking patterns and alcohol-related birth defects. Alcohol Res Health. (2001) 25:168–74. PMC6707176 11810954

[10] Choi KW Abler LA Watt MH Eaton LA Kalichman SC Skinner D. Drinking before and after pregnancy recognition among South African women: the moderating role of traumatic experiences. BMC Pregnancy Childbirth. (2014) 14:97. doi: 10.1186/1471-2393-14-97 24593175 PMC3975846

[11] May PA Hasken JM Blankenship J Marais A-S Joubert B Cloete M. Breastfeeding and maternal alcohol use: Prevalence and effects on child outcomes and fetal alcohol spectrum disorders. Reprod Toxicol (Elmsford NY). (2016) 63:13–21. doi: 10.1016/j.reprotox.2016.05.002 PMC4987236 27174445

[12] Adebiyi BO Mukumbang FC Cloete LG Beytell A-M. Policymakers' Perspectives towards developing a guideline to inform policy on fetal alcohol spectrum disorder: A qualitative study. Int J Environ Res Public Health. (2019) 16:945. doi: 10.3390/ijerph16060945 30884766 PMC6466131

[13] Adebiyi BO Mukumbang FC Cloete LG Beytell AM. Exploring service providers' perspectives on the prevention and management of fetal alcohol spectrum disorders in South Africa: a qualitative study. BMC Public Health. (2018) 18:1238. doi: 10.1186/s12889-018-6126-x 30400900 PMC6220472

[14] Myers B Koen N Donald KA Nhapi RT Workman L Barnett W. Effect of hazardous alcohol use during pregnancy on growth outcomes at birth: findings from a South African cohort study. Alcohol Clin Exp Res. (2018) 42:369–77. doi: 10.1111/acer.13566 PMC5887896 29197115

[15] Marais S Jordaan E Viljoen D Olivier L de Waal J Poole C. The effect of brief interventions on the drinking behaviour of pregnant women in a high-risk rural South African community: a cluster randomised trial. Early Child Dev Care. (2010) 181:463–74. doi: 10.1080/03004430903450392

[16] Rendall-Mkosi K Morojele N London L Moodley S Singh C Girdler-Brown B. A randomized controlled trial of motivational interviewing to prevent risk for an alcohol-exposed pregnancy in the Western Cape, South Africa. Addiction. (2013) 108:725–32. doi: 10.1111/add.12081 23216868

[17] Rotheram-Borus MJ Arfer KB Christodoulou J Comulada WS Stewart J Tubert JE. The association of maternal alcohol use and paraprofessional home visiting with children’s health: A randomized controlled trial. J Consulting Clin Psychol. (2019) 87:551–62. doi: 10.1037/ccp0000408 PMC6775769 31120274

[18] O'Connor MJ Tomlinson M Leroux IM Stewart J Greco E Rotheram-Borus MJ. Predictors of alcohol use prior to pregnancy recognition among township women in Cape Town, South Africa. Soc Sci Med. (2011) 72:83–90. doi: 10.1016/j.socscimed.2010.09.049 21084142 PMC3023822

[19] Onah MN Field S van Heyningen T Honikman S. Predictors of alcohol and other drug use among pregnant women in a peri-urban South African setting. Int J Ment Health Systems. (2016) 10:38. doi: 10.1186/s13033-016-0070-x PMC4855353 27148402

[20] Petersen-Williams P Mathews C Jordaan E Parry CDH. Predictors of Alcohol Use during Pregnancy among Women Attending Midwife Obstetric Units in the Cape Metropole, South Africa. Subst Use Misuse. (2018) 53:1342–52. doi: 10.1080/10826084.2017.1408654 29220610

[21] Morojele NK London L Olorunju SA Matjila MJ Davids AS Rendall-Mkosi KM. Predictors of risk of alcohol-exposed pregnancies among women in an urban and a rural area of South Africa. Soc Sci Med. (2010) 70:534–42. doi: 10.1016/j.socscimed.2009.10.040 19932549

[22] May PA Marais AS Kalberg WO de Vries MM Buckley D Hasken JM. Multifaceted case management during pregnancy is associated with better child outcomes and less fetal alcohol syndrome. Ann Med. (2023) 55:926–45. doi: 10.1080/07853890.2023.2185808 PMC10026770 36919586

[23] de Vries MM Joubert B Cloete M Roux S Baca BA Hasken JM. Indicated prevention of fetal alcohol spectrum disorders in South Africa: effectiveness of case management. Int J Environ Res Public Health. (2015) 13. doi: 10.3390/ijerph13010076 PMC4730467 26703708

[24] Watt MH Eaton LA Choi KW Velloza J Kalichman SC Skinner D. “It's better for me to drink, at least the stress is going away”: Perspectives on alcohol use during pregnancy among South African women attending drinking establishments. Soc Sci Med. (2014) 116:119–25. doi: 10.1016/j.socscimed.2014.06.048 PMC4117814 24997441

[25] Bedwell C Lavender T. Giving patients a voice: implementing patient and public involvement to strengthen research in sub-Saharan Africa. J Epidemiol Community Health. (2020) 74:307. doi: 10.1136/jech-2019-212525 32005647 PMC7079186

[26] Petersen Williams P Petersen Z Sorsdahl K Mathews C Everett-Murphy K Parry CD. Screening and brief interventions for alcohol and other drug use among pregnant women attending midwife obstetric units in cape town, South Africa: A qualitative study of the views of health care professionals. J Midwifery Womens Health. (2015) 60:401–9. doi: 10.1111/jmwh.12328 26220766

[27] Parry CD Gossage JP Marais AS Barnard R de Vries M Blankenship J. Comparison of baseline drinking practices, knowledge, and attitudes of adults residing in communities taking part in the FAS prevention study in South Africa. Afr J Drug Alcohol Stud. (2012) 11:65–76. 25392703 PMC4225715

[28] Tong A Sainsbury P Craig J. Consolidated criteria for reporting qualitative research (COREQ): a 32-item checklist for interviews and focus groups. Int J Qual Health Care. (2007) 19:349–57. doi: 10.1093/intqhc/mzm042 17872937

[29] Braun V Clarke V. Using thematic analysis in psychology. Qual Res Psychol. (2006) 3:77–101. doi: 10.1191/1478088706qp063oa

[30] Lelong N Kaminski M Chwalow J Bean K Subtil D. Attitudes and behavior of pregnant women and health professionals towards alcohol and tobacco consumption. Patient Educ counseling. (1995) 25:39–49. doi: 10.1016/0738-3991(94)00695-I 7603932

[31] Peadon E Payne J Henley N D'Antoine H Bartu A O'Leary C. Women's knowledge and attitudes regarding alcohol consumption in pregnancy: a national survey. BMC Public Health. (2010) 10:510. doi: 10.1186/1471-2458-10-510 20727217 PMC2936428

[32] Kesmodel U Schiøler Kesmodel P. Drinking during pregnancy: attitudes and knowledge among pregnant Danish women, 1998. Alcohol Clin Exp Res. (2002) 26:1553–60. doi: 10.1111/j.1530-0277.2002.tb02455.x 12394289

[33] Watt MH Eaton LA Dennis AC Choi KW Kalichman SC Skinner D. Alcohol use during pregnancy in a South African community: reconciling knowledge, norms, and personal experience. Maternal Child Health J. (2016) 20:48–55. doi: 10.1007/s10995-015-1800-4 PMC4713336 26197733

[34] Fletcher OV May PA Seedat S Sikkema KJ Watt MH. Attitudes toward alcohol use during pregnancy among women recruited from alcohol-serving venues in Cape Town, South Africa: A mixed-methods study. Soc Sci Med. (2018) 215:98–106. doi: 10.1016/j.socscimed.2018.09.008 30219750

[35] Petersen Williams P Jordaan E Mathews C Lombard C Parry CDH. Alcohol and Other Drug Use during Pregnancy among Women Attending Midwife Obstetric Units in the Cape Metropole, South Africa. Adv Prev Med. (2014) 2014:871427–. doi: 10.1155/2014/871427 PMC3930165 24639899

[36] Giglia R Binns C. Alcohol and lactation: A systematic review. Nutr Dietetics. (2006) 63:103–16. doi: 10.1111/j.1747-0080.2006.00056.x

[37] Jones SC Andrews K Francis K. Combining social norms and social marketing to address underage drinking: development and process evaluation of a whole-of-community intervention. PloS One. (2017) 12:e0169872. doi: 10.1371/journal.pone.0169872 28107374 PMC5249059

[38] Wolter C Lesener T Thomas TA Hentschel AC Gusy B. Finding the right balance: A social norms intervention to reduce heavy drinking in university students. Front Public Health. (2021) 9:653435. doi: 10.3389/fpubh.2021.653435 34178916 PMC8222818

[39] Ridout B Campbell A. Using Facebook to deliver a social norm intervention to reduce problem drinking at university. Drug Alcohol Rev. (2014) 33:667–73. doi: 10.1111/dar.12141 24689339

[40] Holder HD Gruenewald PJ Ponicki WR Treno AJ Grube JW Saltz RF. Effect of community-based interventions on high-risk drinking and alcohol-related injuries. JAMA. (2000) 284:2341–7. doi: 10.1001/jama.284.18.2341 11066184

[41] Siriwardhana P Dawson AH Abeyasinge R. Acceptability and effect of a community-based alcohol education program in rural Sri Lanka. Alcohol Alcoholism. (2012) 48:250–6. doi: 10.1093/alcalc/ags116 PMC3571206 23161893

[42] Field S Onah M van Heyningen T Honikman S. Domestic and intimate partner violence among pregnant women in a low resource setting in South Africa: a facility-based, mixed methods study. BMC Women's Health. (2018) 18:119. doi: 10.1186/s12905-018-0612-2 29973182 PMC6030741

[43] Petersen Williams P Brooke-Sumner C Joska J Kruger J Vanleeuw L Dada S. Young South African women on antiretroviral therapy perceptions of a psychological counselling program to reduce heavy drinking and depression. Int J Environ Res Public Health. (2020) 17:2249. doi: 10.3390/ijerph17072249 32230712 PMC7178219

[44] Myers B Carney T Browne FA Wechsberg WM. A trauma-informed substance use and sexual risk reduction intervention for young South African women: a mixed-methods feasibility study. BMJ Open. (2019) 9:e024776. doi: 10.1136/bmjopen-2018-024776 PMC6368003 30782918

[45] Macleod C Matebese S Tsetse N. I drank because I wanted to deal with the frustration": explaining alcohol consumption during pregnancy in a low-resource setting - women's, partners and family members' narratives. Soc Work. (2020) 56:88–96.

